# A189 INVESTIGATING THE ROLE OF BACTERIAL HISTAMINE METABOLISM IN VISCERAL HYPERALGESIA

**DOI:** 10.1093/jcag/gwad061.189

**Published:** 2024-02-14

**Authors:** T Ross, J Pujo, M Hall-Bruce, S Collins, S Vanner, D E Reed, P Bercik, G De Palma

**Affiliations:** Gastroenterology, Farncombe Family Digestive Health Research Institute, Hamilton, ON, Canada; Gastroenterology, Farncombe Family Digestive Health Research Institute, Hamilton, ON, Canada; Gastroenterology, Farncombe Family Digestive Health Research Institute, Hamilton, ON, Canada; Gastroenterology, Farncombe Family Digestive Health Research Institute, Hamilton, ON, Canada; Gastrointestinal Diseases Research Unit, Queens University, Kingston, ON, Canada; Gastrointestinal Diseases Research Unit, Queens University, Kingston, ON, Canada; Gastroenterology, Farncombe Family Digestive Health Research Institute, Hamilton, ON, Canada; Gastroenterology, Farncombe Family Digestive Health Research Institute, Hamilton, ON, Canada

## Abstract

**Background:**

Intestinal microbiota have been implicated in the expression of irritable bowel syndrome (IBS) as patients present with altered gut microbial profiles and microbial metabolic activity. We have previously identified bacterial histamine to strongly influence mast cell accumulation through only IBS activation of the H4 receptor, leading to visceral hyperalgesia in a subset of patients with IBS. We **hypothesize** that a subset of IBS patients with high histamine-producing microbiota exhibit an aberrant histamine metabolism. Investigating the microbiota-driven pathways involved in histamine metabolism is key to understanding abdominal pain pathophysiology in IBS patients.

**Aims:**

1. To study whether variations of histamine levels are due to bacterial metabolism using in vitro and in vivo approaches.

2. To identify the prevalence of high histamine-producing and histamine-degrading bacteria in a clinical cohort via in silico analyses.

**Methods:**

Using in vitro approaches, stool samples from healthy control (HC) donors and IBS patients were inoculated in minimal media in aerobic/anaerobic conditions, with/without excess histidine or added histamine. Bacterial histamine production and degradation were assessed in culture supernatants by ELISA. After identification through Sanger sequencing, individual colony capacity to degrade and produce histamine was assessed. Host and microbial contributions to histamine metabolism will be identified through analyses of germ-free mice colonized with IBS and HC stool samples.

**Results:**

IBS patients (n=23) tested were found to consistently produce higher levels of histamine compared to HC (n=3). Among the tested isolated colonies from IBS patients (n=179) 61% produced histamine compared to 33% of HC (n=54), and 20% degraded histamine compared to 11% of HC. Of these colonies, 13% of only IBS isolates demonstrated the capacity to both degrade and produce histamine. Facultative anaerobes were found to possess both higher production and degradation capacity. Both pH and histamine concentration determine bacterial ability to produce or degrade histamine.

**Conclusions:**

Based on these findings, both healthy and IBS individuals exhibit varying levels of histamine production/degradation, most prominently through facultative anaerobes. The intestinal environment and bacterial community composition are major regulators of bacterial histamine metabolism. The observed in vitro capacity to produce/degrade histamine, and its biological implications, will be confirmed with gnotobiotic humanized mice, ultimately aiding in designing microbiota-directed therapies for the management of visceral hypersensitivity.

**Funding Agencies:**

CIHRFarncombe Innovation Fund

